# Donor orientation and service quality: Key factors in active blood donors’ satisfaction and loyalty

**DOI:** 10.1371/journal.pone.0255112

**Published:** 2021-07-22

**Authors:** Josefa D. Martín-Santana, María Katiuska Cabrera-Suárez, María de la Cruz Déniz-Déniz, Eva Reinares-Lara

**Affiliations:** 1 Department of Economics and Business Administration, Faculty of Economics, Business and Tourism, Universidad de Las Palmas de Gran Canaria, Las Palmas de Gran Canaria, Las Palmas, Spain; 2 Department of Business Economics, Faculty of Legal and Business Sciences, Universidad Rey Juan Carlos, Madrid, Spain; Bucharest University of Economic Studies, ROMANIA

## Abstract

Faced with the increasing demand for blood and greater restrictions on ensuring the safety of transfusions, voluntary donation is currently the only and best alternative for the health system to have a sustainable and safe blood supply. In this context, one of the primary strategies of blood transfusion centres is to increase the satisfaction of their active donors so that they maintain their intention to donate in the future and, in turn, make the necessary recommendations so that third parties can become new donors. That is why this paper raises a question for research concerning, what should the premises be to guide the management of blood transfusion centres to maintain and even increase the satisfaction and loyalty of their active donors? In order to respond to this issue, a change in paradigm is proposed based on a management model supported by donor orientation and service quality as basic pillars, as well as on the development of a number of key social capital resources that explain this orientation toward the donor. In both approaches, the donor becomes the cornerstone of decision-making, moving away from the traditional model which is focused more on achieving targets related to the collection of blood bags. Through the empirical analysis of a sample of 126 employees from various transfusion centres and 26,833 active donors in nine Spanish autonomous communities, we have been able to validate this proposed management model as a useful tool to blood promotion agents in their decision-making processes.

## Introduction

Blood transfusions help save millions of lives every year and ensure that the health system works well, improving patient quality of life and life expectancy by increasing complex interventions in emergency or routine situations. The importance of blood donation is fundamental because (1) blood production in the laboratory is currently impossible, (2) population decline and aging are two factors that negatively affect the balance between supply and demand, (3) the increasing implementation of new donor exclusion measures to ensure safe transfusion and, (4) the voluntary donation system represents the best alternative for the safety and sustainability of the blood supply, since systems based on ‘donation payments’ can present threats to the health and safety of recipients, as well as to the donors themselves [1–3]. Therefore, all countries face the ongoing challenge of obtaining from their donors the blood needed to meet their needs, even more so, given the limited financial resources available to the organisations responsible for promoting donations to attract donors and gain their loyalty [3–5].

In Spain, the blood transfusion centres (CTS by its acronym in Spanish) are health centres responsible for promoting donations. CTS operate in a dual market, since they target not only the beneficiaries of their actions, but also the donors, who play a key role for achieving their goals [6, 7]. These CTS donors or providers of the basic resource (blood) are not only their customers, but their satisfaction with the service makes it possible to support the blood donation system, which would ultimately meet the needs of their beneficiaries (health institutions that receive blood).

According to Álvarez González et al. [6], these types of non-profit organisations, in addition to their external orientation (toward the donor or beneficiary), must also ensure an internal integration and coordination of their departments, units, members and/or employees, a task that falls on those who hold managerial responsibilities. In this regard, the WHO and the IFRC [3] state that, as in any organisation, an effective blood donation system requires effective management. In an organisation with predominantly medical, nursing and laboratory staff, as in most transfusion centres, the importance of these management skills may not be duly recognised or valued sufficiently. However, effective and efficient management in transfusion centres would enable the management team to achieve a stable blood supply, tailored to the needs at every moment, i.e. balancing the demand and supply. This would thus avoid unnecessary donation peaks and, of course, the cost incurred for collecting, testing, treating, and storing blood bags that have an expiration date.

Donors who access CTS altruistically (without financial compensation), may experience risks and/or drawbacks that reduce their desire to remain connected to the donation system. These negative perceptions can be counteracted and overcome both by the intrinsic or extrinsic motivations of the individuals [8, 9] and by the strategies developed by the organisations responsible for blood donation, in which it is necessary to adopt an appropriate management approach [10, 11], thus achieving both the repetition of the donation and its recommendation to other people. Research proposes that the influence of active donors is one of the most effective strategies for recruiting new donors, since current donors can influence their friends, family, co-workers, and other close groups to donate [12, 13]. In addition, being able to rely on regular donors to collect blood and blood components ensures greater blood safety and reduces the collection cost [11].

Therefore, the ultimate objective of the system should be to increase the donor’s loyalty, i.e. the person’s desire to donate again, to donate more, and to recommend his or her friends and family to donate also [14–16]. To this end, it is essential for CTS to provide an optimal service experience to those who come to make a donation, in order to achieve their satisfaction and in this way their loyalty to the system [17].

On the basis of these premises, this paper proposes that there are two basic pillars for the proper CTS management: quality of the service provided to the donor and the adoption by centres of behaviours aimed at their satisfaction. In addition, it is proposed that such focus on the donors will be determined by the development in the CTS of a number of key resources in terms of social capital, such as close coordination among the centres’ departments, the existence of certain shared values regarding the importance of the donor in the system, and the development of trust-building dynamics among its employees.

Thus, this paper proposes a change in paradigm in which the donor becomes the cornerstone of decision-making, moving away from the traditional model which is focused more on achieving targets related to the collection of blood bags.

## Theoretical background

### Service quality as determining factor of donor satisfaction and loyalty

The academic literature on services considers that the interaction of the organisation with its customers is ‘the moment of truth’ or ‘service meeting’. In the donation process, this ‘moment of truth’ refers to the donor’s positive or negative experience with the act of donating, and as a result of this ‘service meeting’, the donor values the service quality and develops a series of attitudes and future behavioural intentions in relation to the organisation [18] that can encourage or discourage such person from donating again [19].

Service quality assesses the level of excellence, that is, the degree to which the service has been provided according to the customer’s expectations [20]. Christopher et al. [21] point out that providing appropriate service quality and meeting or exceeding the customers’ expectations leads to their satisfaction, and the final result of this satisfaction is the likelihood of repeating behaviours and therefore, the recommendation of the service. Service quality as a predictor of customer behaviour has been analysed in both marketing and health literature [22], its components being of an interpersonal, functional, technical, physical and administrative nature [23].

In the case of blood donation, the quality of the donation process should assess the different phases of the donor’s experience in the CTS. Thus, an assessment should be made of aspects ranging from the social and technical skills of the staff, to the condition and design of the facilities, all of which should contribute to the successful donation experience. The tangible aspects of the service (comfort, cleanliness, CTS location…), the professionalism and friendliness of the staff who convey a sense of security and trust to the donor, and the service process itself [24–28], will condition the future behaviour of the donor (repetition, positive mouth-ear response or recommendation to third parties). Thus, it has been found the physical aspects, the design and comfort of the centres and vehicles in which the blood is collected, the attitude and behaviour of the medical personnel, and the service process are the main barriers to continue participating in blood donation [22].

Based on the literature review, it can be concluded that service quality positively influences donor satisfaction and loyalty [20, 29, 30] and that, in order to recruit and retain the donor, CTS should ensure that donors have a satisfactory service experience when they come to donate [31]. If donors undergo a poor service quality experience, this will lead to their dissatisfaction, thus reducing the likelihood that they will donate again [32]. On the contrary, high quality service experience will have a very important effect on donor satisfaction, which will lead to greater commitment [33] and identification with the CTS [34], thus leading them to repeat the experience and therefore recommend it to third parties [12, 13]. In this way, the service quality perceived in previous donation experiences could become a barrier or important motivation to donate in the future [35], which can also influence other donors, both current as well as potential ones, by word of mouth [36]. Therefore, the significance of service quality should be placed in the broader blood donor motivation context, given that the transfusion service quality can play a central role as reciprocal payback, considering that gratitude is predictive of all forms of reciprocity [35]. That is to say, a donor who perceives high quality service will have a sense of gratitude and, therefore, this person will desire to repeat the experience (reciprocity). In summary, high quality service can also be considered a new and less common, yet important, motivation that has not been previously reported as such and can serve as grounds for new recruitment and retention strategies.

Based on the above, the following hypotheses can be established:

***Hypothesis 1*:** *The higher the level of service quality perceived by active donors*, *the stronger their satisfaction*.
***Hypothesis 2*:** *The higher the level of service quality perceived by active donors*, *the stronger their intention to donate blood again*.
***Hypothesis 3*:** *The higher the level of satisfaction of active donors*, *the stronger their intention to donate blood again*.
***Hypothesis 4*:** *The higher the level of satisfaction of active donors*, *the stronger the recommendation of active donors to third parties to donate blood*.
***Hypothesis 5*:** *The higher the level of intention of active donors*, *the stronger their recommendation to third parties to donate blood*.

### Donor orientation and their satisfaction and loyalty

Meeting the needs and interests of donors can be considered the best method of serving the CTS beneficiaries [36], i.e. the health institutions that receive the blood. Therefore, one of the strategies to be followed to achieve donor satisfaction and maintain their loyalty is to have a broad understanding of the influence of marketing and to focus on donors to meet their expectations, thus ensuring the sustainability of the blood supply [37]. Similar to profit-making organisations and based on the seminal work of Balabanis et al. [38], the adoption of a donor orientation philosophy involves (1) generating intelligence about donors and non-donors through formal and informal mechanisms, such as donor surveys, or meetings and discussion spaces with donors and other stakeholders; (2) the dissemination of market intelligence, which refers to the effectiveness with which the organisation communicates the issue among the functional areas of the CTS; and (3) the response, i.e. steps taken to implement action programmes targeting this key stakeholder group (the donors).

Considering the work of Kohli and Jaworski [39], other scholars recognise the positive effects of this focus on customer satisfaction and loyalty [40]. Along the same lines, Sanzo Pérez et al. [41] demonstrate that market orientation has multiple positive effects on customers: retention, satisfaction, added value, loyalty, reduced complaints, and improved image of the organisation. Jaworski and Kohli [42] note that the application of their theoretical framework proposed in Kohli and Jaworski [39] may vary according to the type of organisation on which it is applied, therefore, they point out the need to validate the same in other organisational types, as proposed in this paper when applying it in the non-profit field, more specifically in the CTS.

In this sense, and in the case of non-profit organisations, Duque-Zuluaga and Schneider [43] propose that an orientation toward beneficiaries or users of the service influences the percentage of customers/users who participate/attend/use the services continuously. Along these lines, Miles et al. [44] state that those market-oriented organisations will have more satisfied users that lead them to recommend the service to third parties. Therefore, marketing literature emphasises that the adoption of a market orientation strategy is crucial for most organisations [45]. For non-profit service configurations, which are characterised by direct interaction between customers and the organisation, understanding the effects of implementing market-oriented behaviours is an even greater need [40].

All of the above suggests that a management approach based on donor orientation would favour a change in individual behaviours that would lead to an increase in donor loyalty and, as a result, to the recruitment of new donors. In the case in question, this involves a change of orientation, where the CTS would no longer be so focused on obtaining the largest number of blood bags but on donor satisfaction, i.e. move from production-oriented models to customer-oriented processes based on the provision of quality service [22]. Therefore, the following hypotheses can be established:

***Hypothesis 6*:** *The higher the level of donor orientation*, *the stronger the satisfaction of active donors*.
***Hypothesis 7*:** *The higher the level of donor orientation*, *the stronger the intention of active donors to donate blood again*.

### Internal social capital and donor orientation

The social capital theory is based on the idea that organisations are immersed in relationship networks and, in turn, include relationship networks between individuals and/or groups. In this way, organisations can develop a number of resources based on the existence and quality of the relationships generated in the networks [46, 47]. These social capital resources can also provide access to other resources that organisations need to survive, such as financial resources, knowledge and market access [48]. This paper argues that the existence of high levels of social capital in CTS may be the key to developing a fundamental organisational capacity, such as the ability to target blood donors.

Internal social capital facilitates the existence of dense relationship networks within a social community (e.g. organisation) and generates trust, cohesion and solidarity in the achievement of common objectives. More specifically, three dimensions of social capital can be distinguished: structural, cognitive and relational [47]. The structural dimension refers to the patterns of connection between the stakeholders of a network, i.e. who is connected with who, how these connections occur, and the frequency of information exchange between these stakeholders. The cognitive dimension refers to the common set of objectives, shared vision and common values that are generated as a result of interactions and that promote integration, a sense of shared responsibility and collective action. Finally, the relational dimension includes the resources generated through personal relationships, such as trust, rules and obligations, and identity/identification. Of these resources, this paper focuses on trust as a relational resource, especially relevant for achieving donor orientation.

#### Structural dimension: Interfunctional coordination

Social interactions between the different units of an organisation dilute the boundaries of these units and promote the exchange and recombination of resources (particularly knowledge), the creation and dissemination of innovations, and the formation of common interests [49, 50]. In this way, a rich pattern of relationships and interactions which facilitates the transfer of complex information and tacit knowledge [46, 51, 52] is very important when the information is complex and ambiguous [47], as is the case of market information.

Intense social interaction not only increases the extent and speed of the transfer of information and knowledge among members of a network, but also their expectations that these important resources will be used effectively. In this way, there will be greater motivation to explain, detail or listen to innovative ideas and therefore, there will be greater support and promotion for these initiatives [51, 53]. In other words, social ties can help individuals legitimise their innovative ideas [52]. Organisational creativity and innovation need social interaction and the provision of various resources [52, 54]. In this sense, if groups communicate frequently with different people in other groups, they are more likely to have access to critical resources in terms of instrumental, political, and emotional support. This helps provide support for creative and innovative decisions [55] which can be of great importance when trying to meet customer needs [54] and, for the particular case of non-profit organisations, these can also help meet the needs of donors [36].

A greater intensity of relationships between units can also help in the development of a common language that supports the common knowledge base of the network, which is of great value in achieving shared objectives [47, 56]. This is so, because the existence of a common language avoids misunderstandings and facilitates the resolution of conflicts between members of the network and, in this way, the units will be less absorbed by ’church squabbles’ and more focused on higher-level organisational objectives [46], such as the goal of serving customers (donors) [54].

#### Cognitive dimension: Shared values

A key aspect of the cognitive dimension of social capital needed to achieve donor orientation in the CTS is the extent to which their employees share cultural values of donor orientation. In this sense, general literature on market orientation states that an organisational culture that puts customer interest ahead of those of other stakeholders, such as owners, managers and employees (although not neglecting the interests of these important groups), is a key factor in the development of a long-term profitable organisation [57]. In this way, an organisational culture focusing on these values is considered to be a precedent of market orientation and therefore, the behaviours of this orientation will not appear if the organisation’s culture does not include values that involve a commitment to achieve higher value for customers [58].

This argument can be extrapolated to the CTS context to the extent that a strong donor-oriented culture can be considered as a tool for supporting proper employee behaviour in non-profit organisations [59]. In addition, it can be considered a powerful tool for building a common sense of identity [60]. Likewise, if individuals believe that groups outside the organisation (e.g. blood donors) have a positive image of the organisation, they will tend to identify more strongly with the organisation [61, 62]. This positive assessment of the organisation by customers (donors) will be more likely if the organisational values are oriented toward creating a higher value for them [57].

Therefore, if employees internalise organisational values as part of their own personal concept, they will consider these values as intrinsically motivating [63]. In this sense, there is evidence that if employees identify with the characteristics and values of their organisations, they will be willing to do more to meet the needs of customers and will show higher levels of involvement in their work and excellence in dealing with the customers, thus contributing to the improvement of organisational results [64–67].

#### Relational dimension: Trust

Interpersonal trust constitutes a social capital resource in reference to the extent in which a person is relying on the words, actions, and decisions of another person and is willing to act on the basis of the same [68]. Trust is a key relational resource, because it constitutes a fundamental basis for cooperation, information flow, and knowledge sharing among group members and/or the organisation.

In particular, Lau and Cobb [69] establish that trust is a necessary condition for mutual exchange among colleagues. In this way, trust among members of an organisation can increase the behaviour of organisational citizenship (e.g. providing assistance and collaboration to others beyond what is required by job responsibilities) and interpersonal citizenship behaviour (i.e. supporting and helping colleagues meet their personal goals and the tangible expression of care and concern for them) [68, 70]. This behaviour, which shows generosity, reciprocity, commitment, empathy and cooperation, is an important factor that organisations have to promote if their goal is to develop market orientation [71].

Likewise, a high level of capacity-based trust leads to the development of intimacy and closeness in relationships, and this in turn, can contribute to increased interactions and facilitate the exchange of resources and the effective evaluation of existing information [50]. On the other hand, trust based on people’s concern for others facilitates the acquisition and sharing of new information [72] and encourages organisational creativity. providing an atmosphere in which people are willing to support and follow highly-innovative ideas [73, 74] that can lead to the proposal of novel alternatives [75], thus providing superior value to customers.

Based on the above, the following hypothesis and sub-hypotheses can be established:

***Hypothesis 8*:** *The higher the level of social capital resources at the centres*, *the stronger their donor orientation*:
*Hypothesis 8a*: *The higher the level of interfunctional coordination at the centres*, *the stronger the donor orientation*.
*Hypothesis 8b*: *The greater the consideration of donors as a very important asset of the centre*, *the stronger the donor orientation*.
*Hypothesis 8c*: *The higher the level of interpersonal trust between the employees of the centres*, *the stronger the donor orientation*.

**Fig 1:**
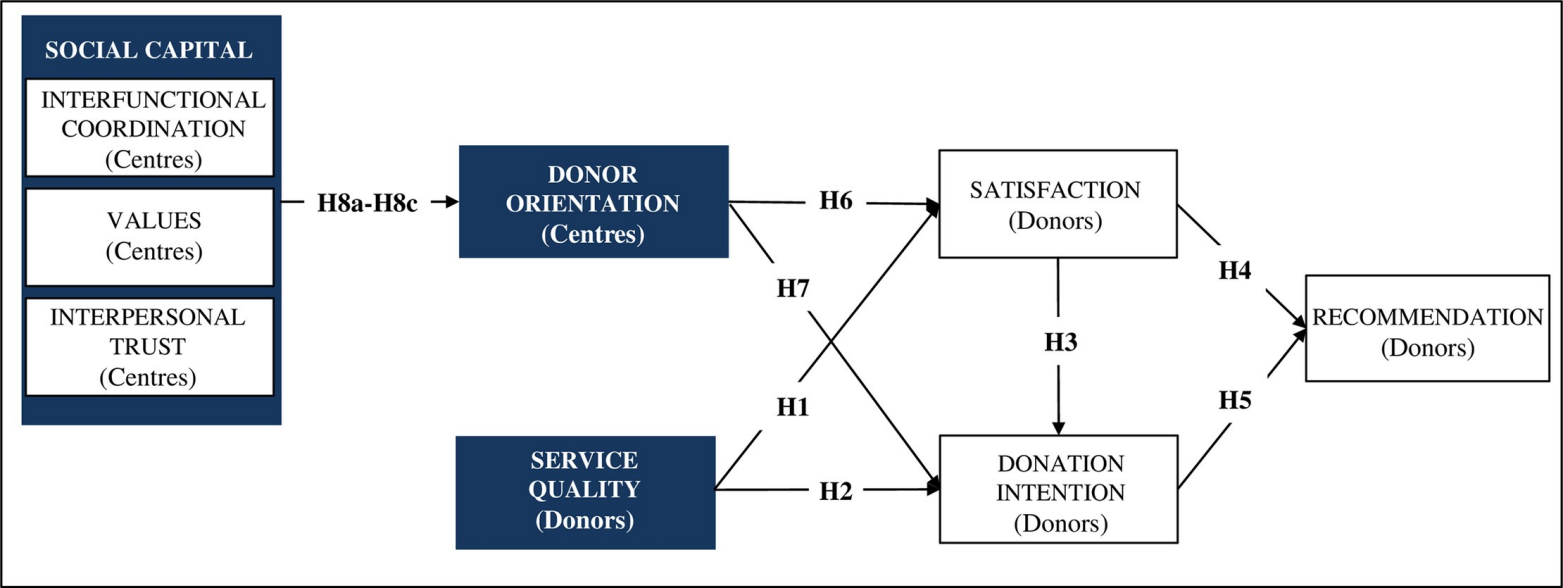
Management model for blood transfusion centres. Note: In each construct, the source of information to be used has been added in parentheses (centre staff or active donors).

The proposed model of relationships between the different constructs analysed in this research is graphically reflected in Fig 1.

## Materials and methods

Two units of analysis are used in this paper. On the one hand, CTS senior and intermediate management team members, as well as the blood collection staff (doctors, nurses and promoters) due to their direct and personal relationship with donors and, on the other hand, the active donors, consisting of those individuals who donated blood at least once in the past two years. The study involved 70.6% of the 17 CTS currently in Spain, which designated a member of their entity as a partner and liaison with the research team. An online questionnaire was used in both study populations to collect the information, and was sent by the CTS partners to staff who were part of the study population and to active donors registered in their databases. The need for more than one response from CTS staff, by having to use aggregated data at CTS level, has forced us to use only information from nine CTS, while the remaining three only gave a single response.

The present study belongs to a nationwide research project supported by the Spanish Ministry of Economy and Competitiveness, which itself constitutes an approval, from an ethical point of view, for its execution. Besides, the project was endorsed by the Spanish Society of Blood Transfusion and Cell Therapy, which is the scientific association of professionals in the field of blood donation and transfusion in Spain. This association actively collaborated throughout the entire project. Lastly, the participation and collaboration of each CTS was expressly authorised by its own internal committee.

Regarding participant consent, participation in this study was completely voluntary, and participants (who were not patients, but 1) individuals registered in the databases of the CTS; 2) members of Spanish university communities—students, teaching and research staff, and administration and services staff -, and 3) CTS employees) could quit the questionnaire any time, without any consequences. For this reason, no written or verbal consent was expressly requested. Additionally, the questionnaire did not include any question which could individually identify the participants. Thus, their responses were completely anonymous.

### Sample profile of CTS staff and of active donors

The sociodemographic profile of the final sample of the CTS staff is shown in Table 1, with the total fully completed questionnaires amounting to 126 and the average completed questionnaires per centre amounting to 14. The sample consists mostly of women (61.1%), aged between 36 and 55 (59.5%), with university studies (70.7%), with time at the centre of less than 10 years (65.1%), and with a stable employment relationship (56.9%).

**Table 1:** CTS staff sample profile.

Characteristics	N	%	Characteristics	N	%
Sex			Seniority at the centre (years)		
Male	49	38.9	0–5	35	27.8
Female	77	61.1	6–10	26	20.6
**Age (years)**			11–15	21	16.7
18–25	5	4.0	16–20	16	12.7
26–35	20	15.9	>20	25	19.8
36–45	34	27.0	**Work relationship**		
46–55	41	32.5	Officer	10	8.1
>55	26	20.6	Permanent statutory staff	16	13.0
**Education**			Temporary statutory staff	27	22.0
Primary	6	4.8	Permanent employment contract	44	35.8
Secondary	30	23.8	Temporary employment contract	26	21.1
University	89	70.7			

The sociodemographic profile of active blood donors collected in the databases of the nine CTS included in the exploitation of the results is shown in Table 2, with the total fully-completed questionnaires amounting to 26,833. This sample is characterised by a balance between both sexes and by being made up of individuals over 35 years of age (63.1%), and with university studies (51.5%). In addition, most participants work and have a monthly income between 1,000 and 4,000 euros.

**Table 2:** Sample profile of CTS active donors.

Characteristics	N	%	Characteristics	N	%
**Sex**			**Working**		
Male	13,010	48.5	Yes.	20,944	78.1
Female	13,823	51.5	No	5,889	21.9
**Age (years)**			**Total monthly income (€)**		
18–25	4,524	16.9	<1,000	3,934	14.7
26–35	5,366	20.0	1,001–2,000	10,598	39.5
36–45	7,495	27.9	2,001–4,000	9,623	35.9
46–55	6.701	25.0	>4.000	2,678	10.0
>45	2,747	10.2			
**Education**					
No education or Primary	3,350	12.5			
Secondary	9,675	36.1			
University	13,808	51.5			

### Measurement scales

All scales used in this study were pretested by 10 CTS officials with the aim of profiling and adapting them appropriately to the context of blood donation. The ‘Donor orientation,’ ‘Interfunctional coordination,’ ‘Shared values’ and ‘Interpersonal trust’ constructs were completed by the participating CTS staff; while the ‘Service quality,’ ‘Satisfaction,’ ‘Donation intention’ and ‘Recommendation’ constructs were completed by the active donors recorded in the CTS databases. The measurement scales used and validated in this work are described below.

#### Donor orientation

This construct was measured by a 23-item, 7-point Likert scale, where 1 meant ‘totally disagree’ and 7 ‘totally agree’. This scale has been designed based on the seminal work of Kohli et al. [76], which has been widely used in the academy for measurement purposes. The three dimensions proposed by these authors were considered: intelligence generation, intelligence dissemination, and response. Some of the items on the original scale had to be adapted or deleted, due to the distinctive characteristics of the organisations responsible for blood donation, as well as the inclusion of new items that would reflect the specific activities of those organisations in their donor management.

#### Interfunctional coordination

The design of this scale has been based on the work of Narver and Slater [57], in which it is established that this construct involves the coordinated use of the organisation’s resources to create a superior value for customers. The final scale used consisted of three items, which were also evaluated through the same 7-point Likert scale reflecting the agreement level.

#### Shared values

The scale used to measure this construct, inspired by the seminal work of Narver and Slater [57], is a two-item, 7-point Likert scale for measuring the level of agreement.

#### Interpersonal trust

A 5-item, 7-point Likert scale, based on the work of McAllister [68], has been used to assess respondents’ trust in the team they usually work with.

#### Service quality

This variable was measured by means of a 17-item, 7-point Likert scale, where 1 represented a ‘very negative assessment’; and 7, a ‘very positive assessment’. This scale was intended to measure several aspects related to the donation centre where the subject usually gives blood. The attributes collected in this scale are based on a review of the literature [32, 77, 78]. The scale consisted of four dimensions: Tangibility, Accessibility, Personal Attention and Professionalism, and Post-Donation. It can be affirmed that this scale represents all the stages of the donation experience, as well as tangible and intangible aspects of the process.

#### Satisfaction

This construct was measured with a 1-item, 7-point Likert scale, where 1 meant ‘completely dissatisfied’, and 7 ‘completely satisfied’, in order to evaluate donor satisfaction with the donation centre. The works of Germain et al. [79] and Morgeson [80] support using a single item to measure this construct. In the context of blood donation, Martín-Santana and Beerli-Palacio [78] used a single attribute to measure donor satisfaction.

#### Donation intention

This construct was measured by a 7-point, two-item Likert scale, based on the work of Boenigk and Helmig [14].

#### Recommendation

This construct was also measured by a 7-point, two-item Likert scale, based on the work of Boenigk and Helmig [14].

The final items used to measure each construct are listed in the S1 Table.

### Validation of the measurement scales

Before the model was tested, the validity of the different measurement scales used in this research was analysed to know its psychometric properties. A confirmatory factor analysis (CFA) was conducted to determine the goodness of fit of each measurement scale. The purpose of this analysis was to test the hypothesis that all items on each scale were measuring a common construct. Because of this, a nonsignificant chi-square valuable is desirable, but since the chi-square statistic is dependent on sample size, a pattern of results across a number of goodness of fit tests was considered. As recommended in the literature [81, 82], these tests were included: Comparative Fit Index (CFI), Normed Fit Index (NFI) and Root Mean Square Error of Approximation (RMSEA). A model is considered to provide better fit to the data as more of the following criteria are met chi-square is nonsignificant, the CFI and NFI are greater than 0.95 and the RMSEA is lower than 0.08. But, values for both the CFI and NFI greater than 0.90 are indicate of acceptable model fit.

In addition, construct validity, which refers to the extent to which a scale provides a reasonable assessment of the construct that it purports to measure, was evaluated calculating the internal consistency, the individual item reliability, the composite reliability and the convergent validity. The internal consistency has been analysed through Cronbach’s alpha coefficient [83], being 0.70 the acceptable value to accept the internal consistency of the scale. The individual reliability of each item on the scale is examined through its factor loading with its construct. As a general rule, it is established that an item can be part of a scale when it presents a factor loading equal to or greater than 0.70, although this rule should not be so rigid, allowing items with lower reliability if their presence improves the validity of the construct [84]. The composite reliability of each scale allows to test, as the Cronbach’s alpha coefficient, the internal consistency of the items of a construct, that is, its capacity to measure a concept together. The acceptable value is 0.70 or higher. The convergent validity of a scale implies that the set of items that form this construct represents the same underlying concept [85], that is to say, all the items tend to measure the same reality and nothing more than that. The analysis of convergent validity is carried out from the mean extracted variance (AVE, Average Variance Extracted) [86], stating that there is convergent validity in a construct when the value of its AVE is greater than 0.50 [87], that is, more than 50% of the variance of the construct is due to its indicators.

The results of these analyses are shown in Table 3. It should be noted that (1) a second-order CFA was applied to validate the Service quality scale and the results point to the existence of four dimensions, which corresponds to those previously established when designing the scale: Tangibility, Accessibility, Personal Attention and Professionalism, and Post-Donation; (2) Donation intention and Recommendation were validated together to avoid adding restrictions to the model, and (3) the analysis of the psychometric properties of the Donor orientation scale was carried out in two stages due to the reduced sample size of the staff of the centres. This limitation has led us to validate each dimension of Donor orientation (DO) firstly, and then the DO construct, based on the results obtained in the CFA for each dimension. To carry out this second stage, we created a new variable for each dimension of DO: Intelligence generation (IG), Intelligence dissemination (ID), and Responsiveness (R). For this purpose, we used a weighted average of the scores given by the respondents to the items that made up each dimension, weighted by the regression weights of each item in the three previous CFA. These variables were labelled with the same name given to each dimension (IG, ID and R).

**Table 3:** CFA for the measurement scales.

Relationships			Individual reliability			Internal consistency			Convergent validity (AVE)
			Standardised estimators	*t*	*p*	Cronbach’s alpha	Composite reliability		
**Intelligence generation (IG)**								
Goodness of fit: *χ*^*2*^ = 35.334, *p* = 0.064, CFI = 0.983, NFI = 0.950, RMSEA = 0.061								
D1_IG	←	**IG**	0.776			0.824	0.860	0.672
D2_IG	←	**IG**	0.875	5.916	0.000			
D3_IG	←	**IG**	0.805	5.734	0.000			
IG1	←	D1_IG	0.815			0.882	0.880	0.712
IG2	←	D1_IG	0.946	9.823	0.000			
IG3	←	D1_IG	0.760	8.212	0.000			
IG4	←	D2_IG	0.966			0.936	0.936	0.881
IG5	←	D2_IG	0.910	12.088	0.000			
IG6	←	D3_IG	0.895			0.949	0.958	0.852
IG7	←	D3_IG	0.949	15.271	0.000			
IG8	←	D3_IG	0.959	14.564	0.000			
IG9	←	D3_IG	0.886	11.781	0.000			
**Intelligence dissemination (ID)**								
Goodness of fit: *χ*^*2*^ = 2.204, *p* = 0.820, CFI = 1.000, NFI = 0.993, RMSEA = 0.000								
ID1	←	**ID**	0.848			0.893	0.897	0.642
ID2	←	**ID**	0.541	5.735	0.000			
ID3	←	**ID**	0.838	10.234	0.000			
ID4	←	**ID**	0.786	9.408	0.000			
ID5	←	**ID**	0.937	11.829	0.000			
**Responsiveness (R)**								
Goodness of fit: *χ*^*2*^ = 57.987, *p* = 0.000, CFI = 0.945, NFI = 0.905, RMSEA = 0.096								
R1	←	**R**	0.892	11.123	0.000	0.929	0.926	0.589
R2	←	**R**	0.553	5.829	0.000			
R3	←	**R**	0.524	5.878	0.000			
R4	←	**R**	0.851					
R5	←	**R**	0.860	10.581	0.000			
R6	←	**R**	0.805	9.683	0.000			
R7	←	**R**	0.857	10.150	0.000			
R8	←	**R**	0.769	8.683	0.000			
R9	←	**R**	0.695	7.390	0.000			
**DONOR ORIENTATION (DO)**								
Goodness of fit: *χ*^*2*^ = 0.856, *p* = 0.355, CFI = 1.000, NFI = 0.995, RMSEA = 0.000								
IG	←	**DO**	0.971			0.946	0.960	0.888
ID	←	**DO**	0.921	14.799	0.000			
R	←	**DO**	0.934	15.099	0.000			
**INTERFUNCTIONAL COORDINATION (COORD)**								
Goodness of fit: *χ*^*2*^ = 0.341, *p* = 0.559, CFI = 1.000, NFI = 0.999, RMSEA = 0.000								
COORD1	←	**COORD**	0.873			0.930	0.921	0.795
COORD2	←	**COORD**	0.911	13.444	0.000			
COORD3	←	**COORD**	0,891	12,435	0.000			
**SHARED VALUES (VAL)**								
Goodness of fit: *χ*^*2*^ = 0.003, *p* = 0.956, CFI = 1.000, NFI = 1.000, RMSEA = 0.000								
VAL1	←	**VAL**	0.958			0.926	0.926	0.863
VAL2	←	**VAL**	0.899	18.868	0.000			
**INTERPERSONAL TRUST (TRUST)**								
Goodness of fit: *χ*^*2*^ = 0.120, *p* = 1.000, CFI = 1.000, NFI = 1.000, RMSEA = 0.000								
TRUST1	←	**TRUST**	0.842			0.923	0.924	0.709
TRUST2	←	**TRUST**	0.867	12.108	0.000			
TRUST3	←	**TRUST**	0.778	10.180	0.000			
TRUST4	←	**TRUST**	0.871	12.187	0.000			
TRUST5	←	**TRUST**	0.850	11.732	0.000			
**SERVICE QUALITY (SERQUAL)**								
Goodness of fit: *χ*^*2*^ = 11.346.761, *p* = 0.000, CFI = 0.957, NFI = 0.957, RMSEA = 0.060								
TANG	←	**SERQUAL**	0.779			0.661	0.798	0.516
ACCE	←	**SERQUAL**	0.893	73.020	0.000			
PA&P	←	**SERQUAL**	0.717	75.532	0.000			
PD	←	**SERQUAL**	0.380	49.831	0.000			
SQ1	←	TANG	0.684	116.104	0.000	0.789	0.827	0.617
SQ2	←	TANG	0.787	134.964	0.000			
SQ3	←	TANG	0.874					
SQ4	←	ACCE	0.548	71.627	0.000	0.714	0.726	0.400
SQ5	←	ACCE	0.639	80.334	0.000			
SQ6	←	ACCE	0.624					
SQ7	←	ACCE	0.709	85.848	0.000			
SQ8	←	PA&P	0.770	115.571	0.000	0.916	0.930	0.659
SQ9	←	PA&P	0.687	104.324	0.000			
SQ10	←	PA&P	0.869	128.379	0.000			
SQ11	←	PA&P	0.895	131.599	0.000			
SQ12	←	PA&P	0.905	132.896	0.000			
SQ13	←	PA&P	0.846	125.507	0.000			
SQ14	←	PA&P	0.674					
SQ15	←	PD	0.545	91.297	0.000	0.807	0.832	0.633
SQ16	←	PD	0.883	138.089	0.000			
SQ17	←	PD	0.906					
**DONATION INTENTION AND RECOMMENDATION (INT and RECOM)**								
Goodness of fit: *χ*^*2*^ = 16.289, *p* = 0.000, CFI = 0.999, NFI = 0.999, RMSEA = 0.024								
INT1	←	**INT**	0.508			0.553	0.601	0.441
INT2	←	**INT**	0.790	30.876	0.000			
RECOM1	←	**RECOM**	0.872			0.852	0.853	0.744
RECOM2	←	**RECOM**	0.853	61.249	0.000			

Table 3 shows the results of the CFA, which indicate, in general, that all the scales used can be considered reliable and valid, since the indexes used are very close to or exceed the thresholds established in the literature. The results of the models showed a suitable fit, since the values of CFI and NFI were higher than 0.95 and the values of RMSEA did not exceed the recommended maximum of 0.08, with the exception of the Responsiveness dimension of the DO. The models demonstrated acceptable individual reliability, since the relationship between each item and its respective dimension/construct was statistically significant, with standardised regression weights higher than or very close to 0.7, and with *t* statistic values also being significant. Some items have been kept on the scales due to content validity, despite their loadings. Also, the internal consistency was estimated by means of the construct reliability. The results show this internal consistency in all cases (see values of composite reliability and Cronbach’s alpha), except for Donation intention. Moreover, the convergent validity was estimated by calculating the AVE. The results indicate that all the critical values were above 0.5, except for the Accessibility dimension of Service quality, and Donation Intention.

## Results

### Comprehensive descriptive analysis of the model constructs

Table 4 shows a descriptive statistic of the different constructs analysed. From a general point of view, it should be noted that in all the constructs and dimensions analysed, the response range of the respondents (staff and donors) is very broad, since in most constructs the minimum is 1 and the maximum is 7. However, mean values and typical deviations reflect that most respondents (staff and donors) score above 4 points, although greater dispersion can be seen in some of them when the standard deviations in value are close to 2. Similarly, Table 4 shows a mean difference test for each construct that indicates the existence or not of differences in the assessments of the respondents according to the autonomous community to which they belong.

**Table 4:** Descriptive statistics of the model constructs.

Construct/ Dimension	Min.	Max.	Mean	SD	Test difference according to Auto. Com. of respondent	
					*F*	*P*
**Donor orientation**	**1.09**	**7.00**	**4.37**	**1.58**	**4.077**	**0.002**
Intelligence generation	1.00	7.00	3.87	1.64	2.298	0.040
Intelligence dissemination	1.00	7.00	4.40	1.84	3.748	0.001
Responsiveness	1.27	7.00	4.89	1.64	3.966	0.001
**Interfunctional coordination**	**1.00**	**7.00**	**4.40**	**1.99**	**3.133**	**0.004**
**Shared values**	**1.00**	**7.00**	**6.12**	**1.40**	**3.704**	**0.001**
**Interpersonal trust**	**1.00**	**7.00**	**5.39**	**1.41**	**2.845**	**0.006**
**Service quality**	**1.00**	**7.00**	**6.11**	**0.73**	**189.434**	**0.000**
Tangibility	1.00	7.00	5.97	1.03	217.525	0.000
Accessibility	1.00	7.00	6.01	0.94	80.675	0.000
Personal Attention and Professionalism	1.00	7.00	6.60	0.64	83.357	0.000
Post-Donation	1.00	7.00	5.69	1.55	465.979	0.000
**Satisfaction**	**1.00**	**7.00**	**6.62**	**0.75**	**29.128**	**0.000**
**Donation intention**	**1.00**	**7.00**	**6.40**	**0.99**	**22.018**	**0.000**
**Recommendation**	**1.00**	**7.00**	**5.97**	**1.37**	**50.879**	**0.000**

In parallel, and in order to quantify the differences between the nine autonomous communities participating in the study in relation to the various constructs, a calculation was made of the mean scores given by the respondents of each community or regional transfusion centre (aggregated scores). These are listed in Table 5. These results reflect again the disparity in management carried out at the centres.

**Table 5:** Mean values of the constructs by autonomous community.

Construct/ Dimension	Autonomous Communities									
		1	2	3	4	5	6	7	8	9
	N_Staff_	18	3	19	6	30	8	2	23	16
	N_Donors_	2,363	2,888	476	2,832	6,040	187	1,998	7,524	2,525
**Donor orientation**		**4.25**	**5.24**	**5.65**	**4.75**	**2.69**	**4.17**	**2.82**	**5.44**	**4.03**
Intelligence generation		3.83	4.28	4.89	4.22	2.53	3.77	2.94	5.02	3.52
Intelligence dissemination		4.63	5.60	4.57	5.60	2.85	5.83	2.18	4.76	5.47
Responsiveness		4.98	5.89	5.37	4.48	3.34	6.16	3.42	6.05	5.15
**Interfunctional coordination**		**4.65**	**5.01**	**4.71**	**3.66**	**3.16**	**4.47**	**2.68**	**5.45**	**5.64**
**Shared values**		**6.28**	**6.84**	**6.87**	**5.83**	**5.12**	**6.63**	**6.50**	**6.16**	**6.60**
**Interpersonal trust**		**5.53**	**5.52**	**5.90**	**5.52**	**4.49**	**5.55**	**4.20**	**5.48**	**6.10**
**Service quality**		**5.75**	**5.97**	**6.25**	**5.90**	**6.15**	**6.18**	**6.43**	**6.19**	**6.17**
Tangibility		5.58	5.99	5.90	5.45	6.04	5.98	6.42	6.11	5.90
Accessibility		5.77	6.11	6.30	5.77	5.96	6.14	6.27	6.07	6.05
Personal Attention and Professionalism		6.48	6.49	6.63	6.46	6.66	6.53	6.81	6.65	6.54
Post-Donation		4.71	4.59	6.13	6.06	5.88	6.04	6.11	5.77	6.32
**Satisfaction**		**6.57**	**6.50**	**6.70**	**6.51**	**6.62**	**6.60**	**6.74**	**6.68**	**6.64**
**Donation intention**		**6.53**	**6.29**	**6.48**	**6.35**	**6.32**	**6.52**	**6.47**	**6.46**	**6.41**
**Recommendation**		**6.32**	**5.82**	**6.09**	**5.83**	**6.01**	**5.86**	**5.96**	**5.84**	**6.25**

As shown in Table 4, the mean of the scores assigned by CTS staff to Donor orientation at their centres reflects a satisfactory level, although with room for improvement (M = 4.37). But at the same time, its standard deviation reflects a certain heterogeneity of the respondents’ perceptions regarding the degree to which donor-oriented behaviours occur in their workplaces (SD = 1.58). This same conclusion is reached when the mean scores of its three dimensions are analysed, ranging from 3.87 to 4.89, the lowest being that obtained in the Intelligence generation dimension, as well as its deviations (ranging between 1.64–1.84), the greatest deviation being in the dimension relative to the Dissemination of intelligence. These differences between the autonomous communities are corroborated by the ANOVA test of differences in means, the results of which indicate that there are statistically significant differences in the perceptions of the centres’ staff in both the overall construct of Donor orientation and in its three dimensions (significance level values less than 5%). The results of Table 5 further highlight the differences between communities or regional centres.

In terms of internal social capital, the average values of their dimensions also show disparity between the CTS staff, since its typical deviations are close to or greater than 1.5 and in addition, the test of difference in means is significant for the three dimensions according to the respondent’s community (significance level values less than 1%). However, it is worth noting the high scores of the cognitive dimension (M = 6.12), followed by the relational dimension (M = 5.39) and, at a great distance, the structural dimension (M = 4.40). The results of Table 5 also indicate the existence of differences between the autonomous communities, with the greatest differences in Interfunctional coordination, ranging from 2.68 to 5.64. The high levels achieved by the cognitive dimension, in which the distances between communities are reduced considerably, ranging from 5.12 to 6.87. This indicates the importance that CTS attaches to donors and the need to achieve their satisfaction.

As can be seen from the data in Table 4, blood donors generally perceive good quality of the donation service, since the overall mean is 6.11 and its deviation is lower than the unit (SD = 0.73). This same conclusion is reached when the mean scores of the four dimensions of Service quality are analysed, the lowest being that obtained in the Post-Donation dimension (M = 5.69) and the highest in the Personal Attention and Professionalism dimension (M = 6.60). However, the results of the ANOVA test indicate that, both in the overall quality construct and in its four dimensions, there are statistically significant differences in donor perceptions according to the autonomous community to which they belong (significance level values less than 1%). The results in Table 5 indicate that there are few differences, since they do not exceed the unit (0.35 and 0.97 points), with the exception of the Post-Donation dimension, where the difference is 1.73 points. These data reflect the important work of the centres in service quality management, which donors are able to perceive.

Finally, as expected in the case of active donors, overall levels of Satisfaction, Donation intention and Recommendation to third parties are very satisfactory (M = 6.62, M = 6.40 and M = 5.97, respectively), although significant differences are observed in terms of the community in which the donors have their place of residence (significance levels less than 1%). The results in Table 5 indicate that there are very few differences, since these do not exceed the unit (0.20, 0.24 and 0.50 points, respectively). These data reflect donors’ commitment to the donation, as well as their work as blood donation prescribers.

### Contrast of the multilevel model

To be able to estimate the multilevel model, it was necessary to incorporate in the active donor database the aggregated scores of the four constructs valued by the staff of the nine autonomous communities (Donor orientation, Interfunctional coordination, Shared values, and Interpersonal trust). To achieve this, all donors in the same community or regional centre were assigned the aggregated scores of their autonomous community or centre. In this way, it was possible to carry out a multi-level study (centres and donors), making it possible to understand how the organisational context of the centres affects the behaviour of their active donors.

**Fig 2:**
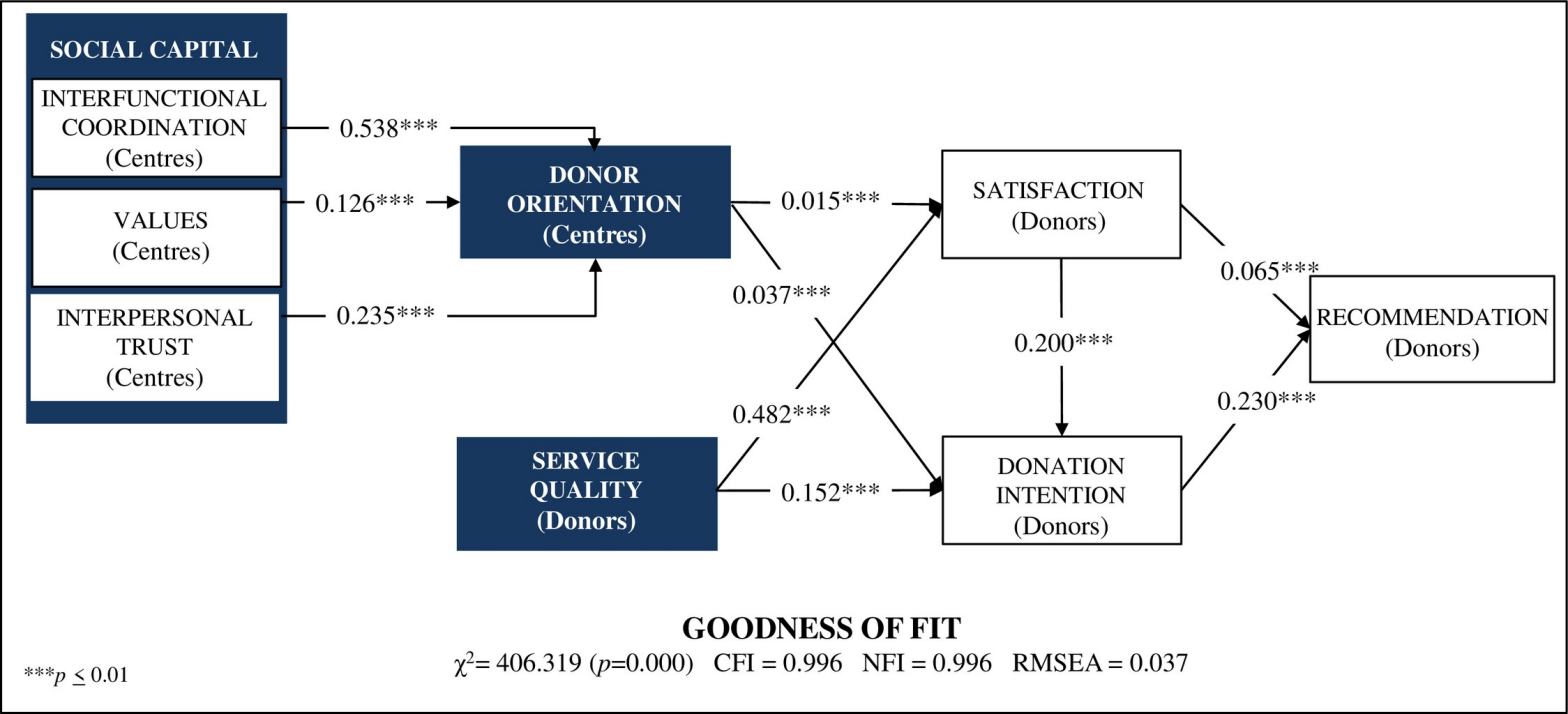
Results of the CTS management model.

To this end, structural equation modelling was used, which makes it possible to know the linear relations between the different constructs included in the model. The results of the same and its adjustment levels are shown in Fig 2, where the levels of the adjustment indicators are satisfactory [*χ*^*2*^ = 406.319, *p* = 0.000; CFI = 0.996; NFI = 0.995, RMSEA = 0.037]. Results in Fig 2 demonstrate that: (1) the three dimensions of social capital are in direct relation to Donor orientation, where Interfunctional coordination is a key to successful implementation of this management approach (*β* = 0.538, *β* = 0.126 and *β* = 0.325), thus accepting H8a, H8b and H8c; (2) both Donor orientation and Service quality management are key elements for Satisfaction and Donation Intention, with Service quality being of greater relevance (*β* = 0.482 and *β* = 0.152 versus *β* = 0.015 and *β* = 0.037), thus giving empirical support to hypothesis H1, H2, H6 and H7; and (3) Satisfaction is a direct antecedent of Donation Intention (*β* = 0.200), thus accepting H3, and both are direct antecedents of Recommendation (*β* = 0.065 and *β* = 0.230), so hypotheses H4 and H5 also obtained empirical support.

## Conclusions

The validation of the proposed multilevel model has made it possible to contrast the suitability and usefulness of a new management paradigm in the CTS, after demonstrating the relationships between the constructs analysed. Thus, it brings to the forefront the importance of proper internal management of CTS focused on donor satisfaction and loyalty. This new management model requires, on the one hand, the enhancement of social capital resources, coordination, shared values and trust, which enable the CTS to achieve the capacity to effectively target donor satisfaction; and, on the other hand, perform their functions with quality levels perceived by donors, satisfying them and motivating them to increase the much-needed loyalty toward donation. Consequently, it is clear that those responsible for CTS management must focus their actions toward a dual objective, which would imply, on the one hand, improve the CTS internal behaviour dynamics by focusing on employees, so that they in turn focus on the donors. On the other hand, it involves improving the tangible and intangible aspects of the donation process (which also depends on employee behaviour) to improve the perception of quality in the donor’s experience. This need to properly manage the CTS human resources may require that their officers (mostly those trained in the health field) receive complementary training in the area of people management. This would include such areas as leadership, motivation and commitment, as well as communication and conflict management dynamics, among others.

If we focus on the two pillars—Donor orientation and Service quality—that, according to the results of this research, support donor satisfaction and loyalty, a number of additional recommendations arise for the CTS. Firstly, and with regard to the dimensions that make up donor orientation, we should consider the lowest mean score of the dimension ‘Intelligence generation’ that, based on the scale proposed in this study to measure the donor orientation, would lead us to recommend that centres update data on: donation evolution, factors that caused inactive donors to stop donating blood, factors that prevent non-donors from donating blood, and actions taken by the blood transfusion centres/services from other Spanish autonomous communities.

Moreover, given the important difference between the centres participating in the study in terms of the efforts that they devote to the dissemination of intelligence, it would be advisable for certain centres to strengthen that dissemination internally and between each other. To this end, the staff responsible for marketing (or similar) activities can hold regular meetings to share and discuss data collected on donors with other divisions or departments, thus ensuring that donor information can be accessed by the staff. Also, they can share information about blood transfusion centres/services from other Spanish autonomous communities. This, in turn would strengthen coordination between areas of the centres and even among centres. This Interfunctional coordination–structural dimension of social capital—has also shown a very low score in this study compared to that of the cognitive and relational dimensions, in which case, structural dimension has been the one that shows great disparity among autonomous communities. Thus, and based on the scale proposed to measure this structural dimension of social capital, members of different areas or departments should interact frequently in order to improve donor recruitment and loyalty, and coordinate activities so as to provide donors with a satisfactory service.

Secondly, the results of this paper show the need for optimal quality in service management in order to achieve satisfaction, donor repetition, and their recommendation to third parties, especially given that the latter has more disparate scores depending on the transfusion centre to which the donor participating in the study belongs. Thus, it is convenient to establish a “culture of service” among the CTS staff [88]. As the data obtained for this study indicate, there is still a wide margin for improvement in the post-donation aspects, where the dimension of Service quality shows great disparity among autonomous communities. This is especially critical because, to some extent, this dimension measures the immediate impact or outcome for the donor, which could increase the value of the service perceived by them. It is therefore recommended that such recognition be made tangible by letters or messages of appreciation, as well as the delivery of useful and easy to understand clinical reports, and suggestions of interest to the donor. Other issues that may be an obstacle to the success of donations are those related with the Tangibility of the service. As the second worst scored dimension of the Service quality, these results lead us to recommend centres to provide facilities that are sufficiently clean, cosy and comfortable, offering privacy during the interview and the donation. All these aspects will have an impact on the perception of quality of the system and in turn on donors’ full satisfaction.

As stated before, the current situation of the CTS indicates that there is room for improvement with regard to donor orientation, the provision of social capital resources, and the quality of service offered. This improvement would require a significant temporary and economic investment that involves various internal divisions in order to achieve, as indicated in the previous paragraphs: a) periodic generation and dissemination of valid and reliable information, b) a strengthening of coordination between areas of the centres and even among the centres, c) an improvement in the post-donation phase, and d) the upgrading of the facilities in which the donation process is carried out. All this will imply at the same time a challenge and great opportunity for the operation of the transfusion centres, allowing them to achieve the necessary donation figures and rates. In line with the proposal of Jaworski and Kohli [42], the tools and scales provided in this research would allow the CTS to distinguish the cost/benefits relation from a transformation toward donor orientation. This would make it possible to balance the supply and demand of blood and avoid the unnecessary donation peaks, as well as the costs of extraction, analysis, treatment and conservation in blood bags subject to an expiration date.

Finally, and with the aim of improving the overall effectiveness of the national blood donation system, it may be advisable to encourage the development of mechanisms for communication and sharing of knowledge, experiences and problems among the CTS officials in the different autonomous communities, so that a common pool of best practices can be developed. In particular, sharing successful marketing experiences that have been developed at certain CTS may be beneficial to the national blood donation system as a whole by reducing the current differences among the various autonomous communities in terms of: dynamics of internal social capital and donor orientation perceived by the CTS employees, experience of donors (service quality), and donors’ satisfaction and loyalty -expressed in terms of intention to donate again and to recommend the donation to third parties-.

Faced with the need to analyse the evolution toward market orientation within the organisations and shed some light on the cultural change process implied thereby [42], a longitudinal assessment -based on the scales developed on this study- could be carried out by CTS in order to monitor their evolution in terms of the management model here proposed. The results obtained so far in this research would constitute a starting point for that longitudinal assessment of the different constructs in the model, since each of the CTS participating in the study received a status report on these variables for their information, and also, as appreciation for their participation in the project. This further research would constitute a practical implementation of the management model proposed in this study and would very likely lead CTS to achieve higher levels of donor satisfaction and loyalty without greater differences according to the autonomous community to which they belong. The strengthening of those aspects in which the centres show greater weakness, together with the effort that the centres themselves and the pertinent authorities in each autonomous community can make to educate the population on the importance of contributing to meeting the blood needs of the health system, would contribute to achieve that necessary balance between the supply and demand of blood.

This research has some limitations. First, it has not delved into the causes behind the differences found among CTS in Spain in relation to each of the constructs in the management model proposed. In depth qualitative studies should be carried out that reveal the cultural causes or other causes of such differences that cannot be detected through a transversal quantitative methodology. Secondly, the generalisation of its results is not possible. Cultural aspects, such as those related with religion or conscience, or institutional aspects, such as the type of organisation that CTS conform in different countries and the regulative environment that dictates the international or regional standards on blood donation could condition the orientation of the CTS to the donors as well as their donors’ satisfaction and loyalty. Therefore, additional research in other cultural or institutional contexts could enhance the generalisation of the results achieved in this research.

## Supporting information

S1 Table Definitive items of the scales. (DOCX)
