# Supplementary material for: Donor orientation and service quality: Key factors in active blood donors’ satisfaction and loyalty
Source: PLoS One. 2021 Jul 22;16(7):e0255112. doi: 10.1371/journal.pone.0255112 (PMC8297764; doi:10.1371/journal.pone.0255112)
Supplement: S1 Table — (DOCX) [file pone.0255112.s001.docx]

| **Construct** | | | | **Code/Items** | | |
| --- | --- | --- | --- | --- | --- | --- |
| **Donor orientation (DO)** | | | | | | |
| **Intelligence generation (IG)** | | | Current donors as a source of information (D1_IG) | IG1 | | Every year we update data on donation evolution, number of donors and donor profiles in detail |
|  |  |  |  | IG2 | | Every year we regularly analyse donor loyalty indicators (lost donors, recovered donors, new donors, retired donors, etc.) |
|  |  |  |  | IG3 | | Every year we regularly distribute a survey to current donors to assess the quality of our services and donor satisfaction |
|  |  |  | Inactive donors and non-donors as sources of information  (D2_IG) | IG4 | | Every year we regularly distribute a survey to inactive donors to know what factors caused them to stop donating blood |
|  |  |  |  | IG5 | | From time to time (no more than every five years) we regularly distribute a survey to non-donors to know what factors prevent them from donating blood |
|  |  |  | Other sources of information  (D3_IG) | IG6 | | Every year regular meetings are held with blood collection staff to gather data about donors |
|  |  |  |  | IG7 | | Every year regular meetings are held between different divisions or departments to analyse data about donors |
|  |  |  |  | IG8 | | We have a fluid relationship with blood transfusion centres/services from other Spanish autonomous communities to share information and experiences |
|  |  |  |  | IG9 | | We often collect and analyse data on actions taken by the blood transfusion centres/services from other Spanish autonomous communities |
| **Intelligence dissemination (ID)** | | | | ID1 | | The staff responsible for marketing (or similar) activities hold regular meetings to share and discuss data collected on donors with other divisions or departments |
|  |  |  |  | ID2 | | Donor information can be accessed by the staff who needs it in accordance with the Spanish Data Protection Act |
|  |  |  |  | ID3 | | Donor information (perceived quality, satisfaction, loyalty, complaints, etc.) is released regularly at every level |
|  |  |  |  | ID4 | | The staff responsible for marketing (or similar) activities is a driver for donation |
|  |  |  |  | ID5 | | Our staff shares and discusses any new and useful information about blood transfusion centres/services from other Spanish autonomous communities |
| **Responsiveness (R)** | | | | R1 | | We use collected data on donors to take actions aimed at improving our results |
|  |  |  |  | R2 | | We offer quick answers to queries and suggestions made by donors through telephone calls, social media, the centre’s website, etc. |
|  |  |  |  | R2 | | Employees are always willing to help donors |
|  |  |  |  | R4 | | We will develop a yearly marketing plan based on professional criteria |
|  |  |  |  | R5 | | When donors demand service improvements, every division or department involved works hard to meet their request |
|  |  |  |  | R6 | | Our staff receives continuous training to provide donors with a top-quality service |
|  |  |  |  | R7 | | We assess the efficiency of donor recruitment and retention plans or programs |
|  |  |  |  | R8 | | We invest a number of resources in developing software to plan, manage and control blood collection |
|  |  |  |  | R9 | | We often carry out joint activities with blood transfusion centres/services from other Spanish autonomous communities aimed at improving donor recruitment and loyalty |
| **Interfunctional coordination**  **(COORD)** | | | | At this centre/service department… | | |
|  |  |  |  | COORD1 | | members of different areas or departments interact often in order to improve donor recruitment and loyalty |
|  |  |  |  | COORD2 | | Activities of different areas or departments are effectively coordinated in order to provide donors with a satisfactory service |
|  |  |  |  | COORD3 | | Every area or department actively participates in devising plans and strategies aimed at increasing blood donation in our organization |
| **Shared values**  **(VAL)** | | | | VAL1 | | We believe that blood donors are the most important resource |
|  |  |  |  | VAL2 | | Keeping blood donors satisfied is a priority |
| **Interpersonal trust**  **(TRUST)** | | | | **In relation to the team that I usually work with, …** | | |
|  |  |  |  | TRUST1 | | we freely share our ideas, feelings and expectations |
|  |  |  |  | TRUST2 | | if I talked to them about my problems, they would respond in a constructive, sympathetic manner |
|  |  |  |  | TRUST3 | | we carry out our jobs professionally and diligently |
|  |  |  |  | TRUST4 | | I trust that none of them will make my job difficult because they will all perform their jobs appropriately |
|  |  |  |  | TRUST5 | | other colleagues whom we interact with trust us |
| **Service quality (SERQUAL)** | **Tangibility**  **(TANG)** | | | SQ1 | The facilities provide privacy during the interview and the donation |  |
|  |  |  |  | SQ2 | The facilities are sufficiently clean |  |
|  |  |  |  | SQ3 | The facilities are cosy and comfortable |  |
|  | **Accessibility**  **(ACCE)** | | | SQ4 | The donation centre or venue (either fixed or mobile) is accessible and easily available |  |
|  |  |  |  | SQ5 | The donation centres or venues’ schedule is convenient |  |
|  |  |  |  | SQ6 | Waiting time before blood collection is half an hour at most |  |
|  |  |  |  | SQ7 | The duration of the donation process is convenient |  |
|  | **Personal Attention and Professionalism**  **(PA&P)** | | | SQ8 | The staff perform well |  |
|  |  |  |  | SQ9 | The staff always explain the requisites to donate, the donation procedure and give recommendations for preventing potential negative effects after donation |  |
|  |  |  |  | SQ10 | The staff are friendly and polite |  |
|  |  |  |  | SQ11 | The staff look after my well-being at all times |  |
|  |  |  |  | SQ12 | The staff inspire confidence during the donation |  |
|  |  |  |  | SQ13 | The staff answer my questions accurately |  |
|  |  |  |  | SQ14 | At the end of the donation, the staff showed their gratitude to me |  |
|  | **Post-Donation**  **(PD)** | | | SQ15 | I get a thank-you letter or message after each donation |  |
|  |  |  |  | SQ16 | The information sent from analysis results is useful |  |
|  |  |  |  | SQ17 | The information that I am sent from analysis results is easy to understand |  |
| **Satisfaction**  **(SAT)** | | | | SAT1 | In general, my level of satisfaction with blood donation at the centre I usually go to is ... |  |
| **Donation intention**  **(INT)** | | | | INT1 | I am going to donate blood in the next four months |  |
|  |  |  |  | INT2 | I would like to become a regular blood donor (twice or more times a year) |  |
| **Recommendation**  **(RECOM)** | | | | RECOM1 | I encourage my relatives, friends and co-workers to donate blood |  |
|  |  |  |  | RECOM2 | I discuss the positive aspects of blood donation among my relatives, friends and co-workers |  |
